# Increased expression of the homologue of enhancer-of-split 1 protects neurons from beta amyloid neurotoxicity and hints at an alternative role for transforming growth factor beta1 as a neuroprotector

**DOI:** 10.1186/alzrt134

**Published:** 2012-07-31

**Authors:** Pedro J Chacón, Alfredo Rodríguez-Tébar

**Affiliations:** 1 Centro Andaluz de Biología Molecular y Medicina Regenerativa (CABIMER), Americo Vespucio s/n, Isla de la Cartuja, 41092 Seville, Spain

## Abstract

**Introduction:**

Alzheimer's disease (AD) is a neurodegenerative disorder characterized by the deposition of β-amyloid (Aβ) in the brain, which produces progressive neuronal loss and dementia. We recently demonstrated that the noxious effects of Aβ on cultured hippocampal neurons are in part provoked by the antagonism of nerve growth factor (NGF) signalling, which impairs the activation of nuclear factor κB (NF-κB) by impeding the tyrosine phosphorylation of I-κBα. As a result, the expression of the homologue of Enhancer-of split 1 (*Hes1*) gene is downregulated and ultimately, gamma-aminobutyric acid (GABA)-ergic connectivity is lost.

**Methods:**

*Hes1 *activity was promoted in cultured hippocampal neurons by overexpressing a *Hes1*-encoding plasmid or by upregulating this gene by activating NF-κB through different approaches (overexpressing either the I-κB kinaseβ, or p65/RelA/NF-κB). Alternatively neurons were exposed to TGFβ1. Dendrite patterning, GABAergic connectivity and cell survival were analyzed by immunofluorescence microscopy. *Hes1 *expression was determined by real-time PCR. NF-κB activation was measured using the dual-luciferase reporter assay.

**Results:**

The expression of *Hes1 *abolished the effects of Aβ on dendritic patterning and GABAergic input, and it prevented the death of the cultured neurons. TGFβ1, a known neuroprotector, could counteract the deleterious effects of Aβ by inducing NF-κB activation following the serine phosphorylation of I-κBα. Indeed, the number of GABAergic terminals generated by inducing *Hes1 *expression was doubled.

**Conclusion:**

Our data define some of the mechanisms involved in Aβ-mediated cell death and they point to potential means to counteract this noxious activity.

## Introduction

The amyloid hypothesis considers amyloid beta (Aβ) as the principal agent underlying the various manifestations of Alzheimer's disease (AD). Accordingly, most therapeutic approaches for AD treatment target the Aβ peptide, which induces the formation of amyloid deposits in the brain [1]. These strategies attempt to limit Aβ production and fibril formation, or to increase Aβ clearance from brain deposits. This latter approach has been the focus of much investigation, with active and passive methods to immunize against Aβ effectively reducing brain Aβ content. However, this reduction in amyloid content has not been conclusively associated with improvements in cognitive performance or a slowing in the progression of AD [2,3].

An alternative approach to AD therapy involves interfering with the signals transduced by Aβ to offset neuronal deterioration and death. While neuronal Aβ signaling pathways have been studied extensively, many of the underlying mechanisms remain elusive. Several studies have related Aβ neurotoxicity with NGF neurotrophy and indeed, some current therapeutic approaches for AD involve the use of NGF or other compounds that mimic its effects [4-9]. The cellular and molecular bases underlying the antagonism of NGF by Aβ have been described recently, at least in part. Accordingly, Aβ competes with nerve growth factor (NGF) binding to p75^NTR ^[10,11], thereby preventing the activation of NF-κB by impairing the tyrosine phosphorylation and the subsequent degradation of I-κBα [12]. The inhibition of nuclear factor kappa-B (NF-κB) mediated by Aβ results in the downregulation of the *enhancer of split homolog 1 *(*Hes1*) gene, which is known to influence dendrite patterning and gamma-aminobutyric acid (GABA)-ergic inputs [13,14].

We recently reported that Aβ impairs the initial steps of NGF signalling at the level of RhoA GTPase and protein tyrosine phosphatase 1B (PTP1B) [15]. Furthermore, we demonstrated that potentiation of NGF signalling (for example, by inhibiting RhoA GTPase and activating PTP1B) confers neuronal resistance to Aβ neurotoxicity. In the present study, we explored alternative means of activating NF-κB and promoting *Hes1 *expression. We found that overexpression of I-κβ kinase (IKKβ) a kinase that phosphorylates I-κBα (an NF-κB inhibitor), p65/RelA or *Hes1 *abolished the effects of Aβ on dendritic patterning, GABAergic input and the survival of cultured hippocampal neurons. Furthermore, administration of transforming growth factor β1 (TGFβ1) produced similar effects, augmenting *Hes1 *expression and GABAergic input, and conferring resistance to Aβ toxicity. These results further our understanding of Aβ toxicity in AD and they open new perspectives for AD treatment using anti-amyloid approaches.

## Materials and methods

### Antibodies

The following primary antibodies were used for immunocytochemistry: rabbit anti-enhanced green fluorescent protein (EGFP, 1:500: Invitrogen, Carlsbad, CA, USA), chicken anti-EGFP (1:500: Chemicon, Hampshire, UK), mouse anti-Myc (1:400: Roche Applied Science, Mannheim, Germany), mouse anti-FLAG (1:500: Sigma-Aldrich, Madrid, Spain) and rabbit anti-vesicular inhibitory amino acid transporter (VIAAT, 1:400: Chemicon). The following secondary antibodies were used: goat anti-rabbit Cy^2 ^(1:1000), goat anti-mouse Cy^3 ^(1:1000), goat anti-rabbit Cy^3 ^(1:1000) and goat-anti mouse Cy^5 ^(1:500: all from Jackson Immuno Research, West Grove, USA), and goat anti-chicken Alexa-fluor 488 (1:1000: Invitrogen).

### Amyloid β preparation and characterization

Amyloid β (1-42) was purchased from NeoMPS (Strasbourg, France) and to obtain oligomeric forms, the peptide was dissolved in 1,1,1,3,3,3-hexafluoro-2-propanol and the solution was allowed to evaporate for 2 h at room temperature. The peptide film was dissolved in dimethyl sulfoxide (DMSO), sonicated in a water bath for 10 minutes and diluted to 100 μM in PBS. This solution was then briefly vortexed and incubated overnight at 4°C. Aliquots were stored at -20°C. Our Aβ preparations were resolved by 12% Bis-Tris gel electrophoresis and electrotransferred to polyvinylidene difluoride (PVDF) membranes, which were probed with a mouse monoclonal anti-β Amyloid antibody (clone BAM-10, diluted 1:2,000: Sigma Aldrich). After further incubation with an horseradish peroxidise (HRP)-conjugated anti-mouse antibody (1:10,000: Jackson Immuno Research, West Grove, USA), immunoreactive Ab species were visualised by chemiluminiscence. This analysis revealed that most Aβ forms were monomeric and dimeric with a less prominent trimeric and tetrameric component (Figure S1A in Additional file 1). To kill neurons in culture, a concentration of 5 μM was established empirically (Figure S1B in Additional file 1).

### Other chemicals

Recombinant TGFβ1 was purchased from Preprotech EC LTD (London, UK). The cell-permeable NF-κB inhibitor peptide SN-50 and its inactive control, SN-50M, were obtained from Calbiochem (Darmstadt, Germany).

### Expression vectors for neuronal transfection

EGFP-expressing vector (pEGFP-N1) was obtained from Biosciences/Clontech (Palo Alto, CA, USA). Vectors expressing the wild type (wt) forms of IKKα (pCDNA-HA-IKKα) and IKKβ (pRK5cFLAG-IKKβ) were kindly provided by Lisardo Boscá [16]. Vectors encoding mutant forms of I-κBα (3XFLAG-pCMV-I-κBαS32/36A and 3XFLAG-pCMV-I-κBαY42F) were described previously [17]. A FLAG-tagged vector encoding p65/RelA (pCMV-Tag1-p65) was kindly provided by Mayte Coiras [18]. Vectors encoding *Hes1 *(pCLIG-Hes1) and *Hes6 *(pIRES-Hes6) were kindly provided by Ryoichiro Kageyama [19] and Phil Jones [20], respectively. Myc-tagged forms of Hes1 and Hes6 were obtained by inserting the corresponding cDNA encoding sequence from previous vectors into the NcoI/EcoRI and NcoI/StuI sites of the pCS2+MT vector.

### Cell culture

Primary hippocampal neuronal cultures were prepared as described previously [21], with some modifications. Briefly, hippocampi were dissected from embryonic day 17 (E17) CD1 mouse foetuses and dissociated into single cells following trypsin digestion (Worthington, Lakewood, USA) and DNase I treatment (Roche Applied Science). Neurons were plated on glass coverslips or in plastic dishes coated with poly-L-lysine (Sigma-Aldrich, Madrid, Spain), and then cultured in Neurobasal A supplemented with 2 mM GlutaMAX I, 100 units/mL penicillin and 100 μg/mL streptomycin (Gibco BRL, Crewe, UK). After 7 days *in vitro *(DIV) the neurons were exposed to either TGFβ1 or Aβ.

### Neuronal transfection

For fluorescence immunocytochemistry, cultured neurons (7 days *in vitro*, DIV) were transfected with different vectors using the Effectene Transfection Reagent (Qiagen GmbH, Hilden, Germany) according to the manufacturer's instructions, with some modifications. Briefly, 0.6 μg of DNA was added to 120 μl of EC buffer and 3.5 μL of enhancer, and then left for 5 minutes at room temperature before 10 μL of Effectene was added. After 15 minutes incubation at room temperature, the final solution was added to a 35 mm cell culture dish containing hippocampal neurons, for 3 h before the medium was changed. Less than 0.5% of the neurons were transfected in each dish, permitting the morphology of the neurons to be analyzed without the interference of neighboring labeled neurons. To determine *Hes1 *mRNA, 300,000 neurons were transfected with different vectors using Lipofectamine LTX (Invitrogen), following the protocol recommended by the manufacturer. The rate of transfection was 20-25% of the total number of cells.

### Immunocytochemistry, image acquisition and morphometric analysis of labeled hippocampal neurons

Neurons were fixed for 30 minutes in 4% paraformaldehyde (PFA) in PBS 18 h after transfection, permeabilized for 15 minutes at room temperature with 0.5% Triton X-100 in PBS and blocked with 10% goat serum in PBS containing 0.1% Triton X-100. The neurons were then incubated with the primary and secondary antibodies. To verify that the labeling was caused specifically by the primary antibodies, it was either omitted or replaced by similarly diluted normal serum from the same species.

Our methods for the evaluation of dendritic morphology and presynaptic terminal identification in dissociated cell cultures have been described previously [14]. Briefly, labeled neurons were visualized by standard epifluorescence under a Plan-Neofluar 63× oil objective with a numerical aperture of 1.3 (Zeiss, Oberkochen, Germany). Terminal counts and analysis of dendrite morphology were performed manually. A circular region of interest (ROI) with a diameter of 50 μm was projected onto the EGFP-labeled neuron, its centre roughly coinciding with the centre of the soma. The dendrite length was expressed as the number of dendritic trees that exceeded the limit of the ROI (number of dendrites > 50 μm). In co-transfection experiments, only double-labeled cells were analysed, representing > 90% of the single-labeled cells. Synaptic terminals in contact with an EGFP-labeled neuron were identified by single immunofluorescence, when an antibody against VIAAT was used.

### Cell survival

After treatment, neurons were fixed for 30 minutes in 4% paraformaldehyde (PFA) in PBS and the nuclei were immunostained with the fluorescent dye 4',6-diamidino-2-phenylindole (DAPI: Sigma-Aldrich). Non-viable neurons were recognized by nuclear condensation and/or fragmented chromatin. In two independent experiments, the number of viable neurons was counted in triplicate in approximately 50 fields. In co-transfection experiments, only the nuclei of double-labeled cells were analysed.

### Quantitative real time polymerase chain reaction (PCR)

After treatments, total RNA was extracted from cultures at 7 DIV using the Illustra RNAspin Mini kit (GE-Healthcare, Piscataway, NJ, USA) and first strand cDNA was prepared from the RNA using the First Strand Synthesis kit according to the manufacturer's instructions (Fermentas GmbH, St Leon-Rot, Germany). Quantitative PCR was performed using the ABI Prism 7000 Sequence Detector (Applied Biosystems, Weiterstadt, Germany). TaqMan probes and primers for *Hes1 *and the housekeeping gene *GADPH *(as a control) were selected as the Assay-on-Demand gene expression products (Applied Biosystems). All TaqMan probes were labeled with 6-carboxy fluorescein (FAM) and real time PCR was performed using the TaqMan Universal PCR Master Mix according to the manufacturer's instructions. *Hes1 *expression was normalized to *GAPDH *expression.

### Analysis of reporter gene activity

For reporter gene studies, 12-well plates (Becton Dickinson) containing 200,000 hippocampal neurons were transfected using the Lipofectamine LTX (Invitrogen) transfection reagent according to the manufacturer's instructions. Transfection efficiency ranged from 20 to 25% in control experiments, as revealed by co-transfection with pEGFP-N1 (Biosciences/Clontech). Cells were co-transfected with 0.5 μg/well of a plasmid containing five tandem repeats of the κB enhancer element upstream of the coding sequence of firefly-luciferase (pNF-κB-luc; Clontech), along with 0.1 μg of a plasmid encoding Renilla-luciferase (phRL-TK-luc: Promega, Madison, WI) as a transfection control for normalization in dual-luciferase assays. Dual-Luficerase reporter assays were performed according to the manufacturer's recommendations (Promega).

### Statistical analysis

The data are presented as the mean ± standard error of the mean (SEM). Unpaired Student's *t*-tests were used to determine the significance, denoted as *P < 0.05, **P < 0.01, and ***P < 0.001. All experiments were repeated at least twice.

## Results

### Hes1 in neuronal morphology, connectivity and survival

**Figure 1:**
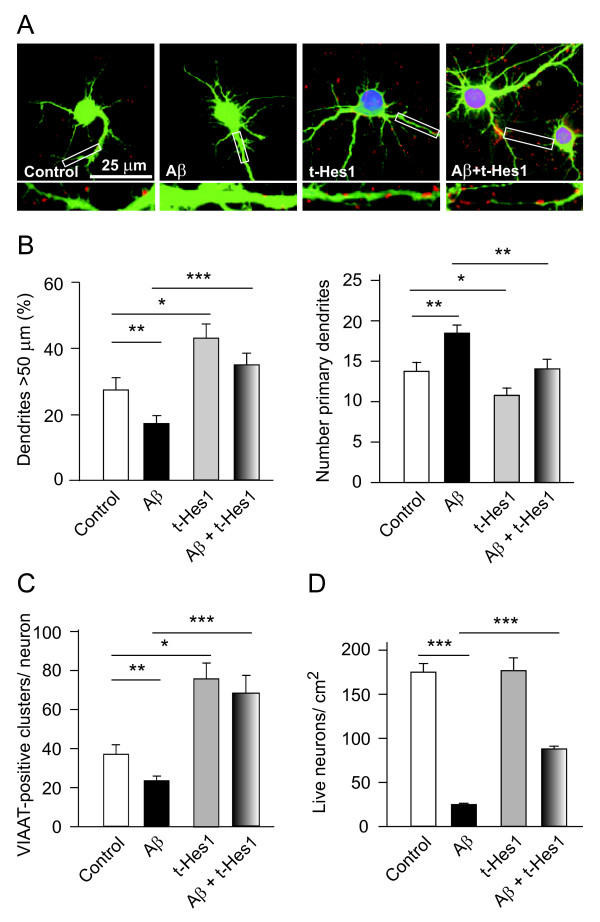
***Hes1 *overexpression blocked the effects of amyloid beta (Aβ) on the morphology, GABAergic connectivity and survival of cultured hippocampal neurons**. (**A-C**) E17 hippocampal neurons were plated at a density of 40,000 cells/cm^2 ^and cultured for 7 days *in vitro *(DIV). Neurons were treated with Aβ (5 μM), and/or they were co-transfected with enhanced green fluorescent protein (EGFP) and a myc-tagged *Hes1 *vector for 16 h. The cells were fixed and labeled with anti-EGFP, anti-myc and anti- vesicular inhibitory amino acid transporter (VIAAT) antibodies, and examined by immunofluorescence. (**A**) Representative micrographs of cultured neurons under different conditions. Nuclear labeling (in purple) corresponds to myc-tagged *Hes1*. Punctuated red dots correspond to VIAAT immunostaining. Lower panels show the boxed regions at higher magnification. (**B**) Morphometric analysis was performed as indicated in Materials and methods. Note that Aβ decreased the length of primary dendrites (left panel) and increased their number (right panel), whereas *Hes1 *induced opposite effects, counteracting those of Aβ. (**C**) *Hes1 *overexpression increased the number of GABAergic terminals in cultured neurons, overriding the decrease induced by Aβ administration. (**D**) 7 DIV neurons (30,000 cells/cm^2^) were treated with Aβ (5 μM). Two days later the neurons were co-transfected with EGFP and the myc-tagged *Hes1 *vector, and the cells were stained with anti-EGFP, anti-myc antibodies and 4',6-diamidino-2-phenylindole (DAPI) on the following day. EGFP and myc dual-labeled cells with intact nuclei were counted. Approximately 50% of transfected neurons survived Aβ treatment. *P < 0.05, **P < 0.01, and ***P < 0.001.

Earlier studies revealed that *Hes1 *mediates the effects of NGF on neuronal morphology and connectivity. NGF increases both dendrite length and GABAergic connectivity in cultured hippocampal neurons, effects that are abrogated by overexpression of Hes6 [13,14], a natural inhibitor of Hes1 [19]. We transfected a *Hes1*-expressing vector into cultured hippocampal neurons and analyzed the effects on dendrite morphology and synaptic connectivity. Overexpression of *Hes1 *increased the length of primary dendrites while decreasing their number (Figure 1A, B), and it enhanced GABAergic connectivity, as evident by immunostaining for VIAAT-positive clusters (Figure 1C). Interestingly, *Hes1 *transfection of neurons protected these cells from the neurotoxic effects of Aβ. Indeed, while oligomerization of Aβ (Figure S1A in Additional file 1) decreased the length and increased the number of primary dendrites, albeit decreasing GABAergic input, these effects were completely reversed by *Hes1 *transfection (Figure 1A, B and 1C). Moreover, *Hes1 *overexpression attenuated the effects of Aβ on neuronal survival. Using a concentration of 5 μM Aβ(1-42) in these studies to ensure that most cells would die during the experiment (performed over 90 h (Figure S1B in Additional file 1), prior transfection with *Hes1 *rescued 50% of neurons from this Aβ-induced death (Figure 1D). Taken together, these findings demonstrate that optimal expression of *Hes1 *counteracts the effects of Aβ on neurons at all the levels examined here.

### Activation of NF-κB blocks the effects of Aβ on neuronal morphology and connectivity

**Figure 2:**
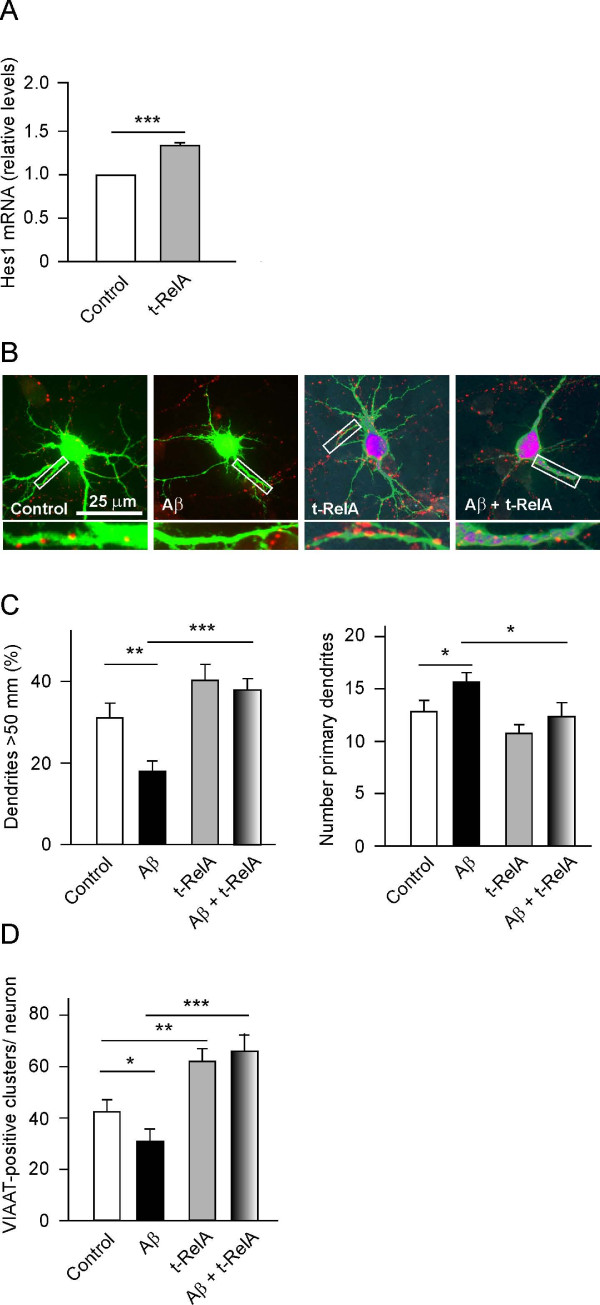
**Transfection of p65/RelA promoted Hes1 expression and counteracted the effects of amyloid beta (Aβ) on the morphology and GABAergic connectivity of cultured hippocampal neurons**. (**A**) 7 days *in vitro *(DIV) neurons (300,000 cells) were transfected with a plasmid encoding myc-tagged p65/RelA. After 16 h, the cells were lysed and processed for real time-PCR, showing that *Hes1 *expression levels increased by 35%. (**B-D**) Cultured hippocampal neurons (40,000 cells/cm^2^, 7 DIV) were co-transfected with enhanced green fluorescent protein (EGFP) and myc-p65/RelA plasmids, treated with Aβ (5 μM) and incubated for a further 16 h, before analyzing dendritic patterning (**B, C**) and GABAergic connectivity (**B, D**). (**B**) Representative micrographs of 7 DIV hippocampal neurons treated with Aβ and/or transfected with p65/RelA. EGFP immunostaining (green) and the transfected p65/RelA mostly located to the nucleus (purple). Vesicular inhibitory amino acid transporter (VIAAT) was evident as punctuated red dots. Lower panels show the boxed regions at higher magnification. (**C**) Morphometric analysis of treated neurons. p65/RelA overexpression increased the length (left panel) but decreased the number (right panel) of primary dendrites, thereby counteracting the effects of Aβ. (**D**) p65/RelA overexpression increased the number of GABAergic terminals in cultured neurons and overrode the decrease in GABAergic terminals produced by Aβ. *P < 0.05, **P < 0.01, and ***P < 0.001.

Having previously revealed that NGF augments *Hes1 *by activating NF-κB [13], the link between NF-κB activation and *Hes1 *expression was confirmed here using an alternative experimental approach. Cultured hippocampal neurons were transfected with a plasmid expressing p65/RelA, and *Hes1 *expression was assessed by quantitative PCR. Although only 20 to 25% of neurons were transfected, a significant increase (35 to 40%) in *Hes1 *mRNA was evident throughout the culture (Figure 2A). Transfected, myc-tagged p65/RelA was predominantly found in the nucleus, as expected given its capacity to promote *Hes1 *expression (Figure 2B). Moreover, p65/RelA overexpression produced marked changes in dendrite arborisation, increasing the length and decreasing the number of primary dendrites (Figure 2B). These effects were paralleled by a substantial increase in GABAergic connectivity (Figure 2B, D) concomitant with an increase in *Hes1 *expression (Figure 1). As seen following *Hes1 *transfection, p65/RelA transfection blocked the effects of Aβ on both dendrite morphology and connectivity, preventing the increase in dendrite length and the decrease in dendrite number induced by Aβ (Figure 2A, B and 2C). Indeed, p65/RelA overexpression also prevented the decrease in VIAAT-positive terminals induced by Aβ. The anti-amyloid effects of p65/RelA overexpression on neuronal survival could not be studied, as p65/RelA overexpression induced neuronal death two days after transfection (that is, before Aβ had noxious effects on cultured neurons).

### IKKβ activation counteracts the effects of beta-amyloid

**Figure 3:**
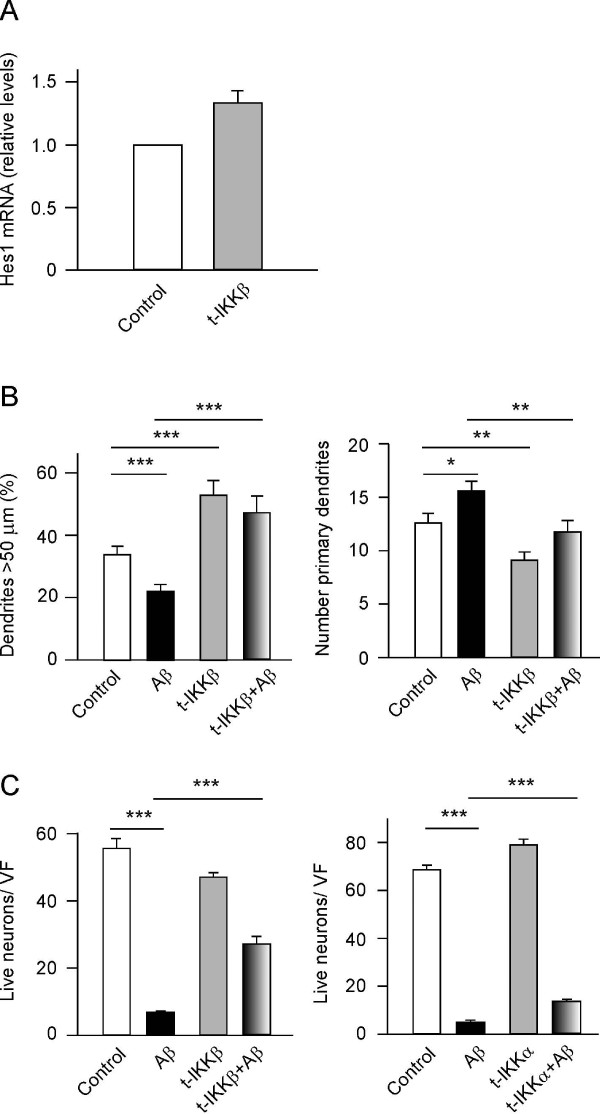
**I-κB kinase (IKK-β) induces *Hes1 *expression, modulates dendritic morphology and neuronal survival, and it counteracts amyloid beta (Aβ) activity**. (**A**) The efficiency of transfecting 7 days *in vitro *(DIV) cultured neurons (300,000 cells) with a vector expressing Flag-tagged IKKβ was 20-25%. After 16 h, the cells were lysed and *Hes1 *expression was quantified by real time PCR, and transfection of IKKβ increased *Hes-1 *mRNA levels by 35%. (**B**) Hippocampal neurons were cultured for 7 DIV (40,000 cells/cm^2^), treated with Aβ (5 μM), and/or co-transfected with enhanced fluorescent green protein (EGFP) and FLAG-tagged IKK-β for 16 h. Transfection with IKKβ increased the length (left panel) but decreased the number (right panel) of primary dendrites, and blocked the effect of Aβ on dendrite morphology. (**C**) 7 DIV hippocampal neurons (30,000 cells/cm^2^) were cultured for 7 days and then treated with Aβ (5 μM). Two days later, the neurons were co-transfected with EGFP, FLAG-tagged IKKβ and/or HA-tagged IKKα and the number of live cells was determined on the following day. IKKβ transfection rescued about 50% of neurons from Aβ-induced death (left panel), whereas this rescue was more modest when neurons were transfected with IKKα rather than IKKβ (right panel). *P < 0.05, **P < 0.01, and ***P < 0.001.

NGF was shown to activate NF-κB via tyrosine phosphorylation and the subsequent degradation of I-κBα [12]. The canonical NF-κB activation pathway involves Ser(32,36) phosphorylation of I-κBα catalysed by the IKKs, and hence we tested whether increased activity of IKK proteins conferred amyloid resistance to cultured neurons. When cultured hippocampal neurons were transfected with a plasmid overexpressing IKKβ, again only 20 to 25% of neurons were transfected (data not shown). Nonetheless, the levels of Hes1 mRNA increased significantly (25 to 30%) throughout the entire culture (Figure 3A). As expected, IKKβ overexpression produced similar alterations in neuronal morphology as *Hes1 *or p65/RelA transfection (longer and fewer dendrites) (Figure 3B). In addition, IKKβ transfection conferred hippocampal neurons with resistance to Aβ (Figure 3C, left panel). These effects were specific to IKKβ as transfection with IKKα produced no noticeable changes in dendrites (not shown) and conferred only very modest resistance to Aβ neurotoxicity (Figure 3C, right panel).

### Effects of TGFβ1 on neuronal morphology, connectivity and survival

**Figure 4:**
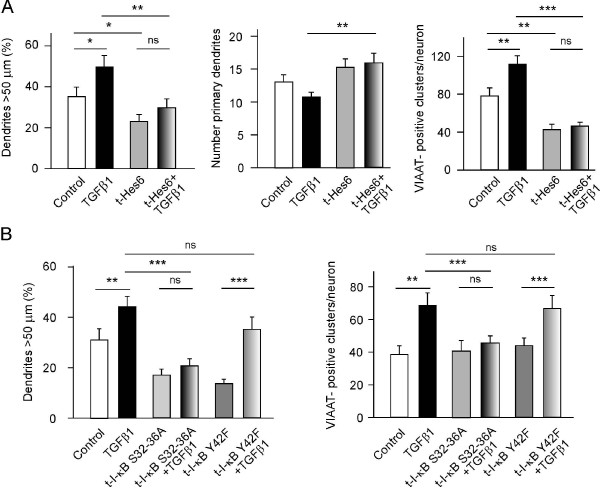
**Transforming growth factor β1 (TGFβ1) controls dendrite morphology and neuronal connectivity via I-κBα and *Hes1***. Hippocampal neurons (7 days *in vitro *(DIV), 40,000 cells/cm^2^) were treated with TGFβ1 (10 ng/ml) before they were co-transfected with pEGFP and plasmids overexpressing either myc-tagged Hes6 (**A**) or mutant forms of FLAG-tagged I-κBα (**B**) for 16 h. The neurons were then processed for immunocytochemistry using anti-EGFP and anti-myc/anti-FLAG antibodies. (**A**) Overexpression of *Hes6*, a known inhibitor of *Hes1*, abrogated the effects of TGFβ1 on dendritic length (left panel) and on the number of primary dendrites (middle panel). *Hes6 *overexpression also prevented the TGFβ1-induced increase in the number of GABAergic terminals (right panel). (**B**) Transfection of I-κBα S32-36A (a mutation that affects serine phosphorylation of the inhibitor) prevented the TGFβ1-induced increase in dendritic length (left panel) and attenuated the formation of vesicular inhibitory amino acid transporter (VIAAT)-positive clusters (right panel). The I-κBα Y42F mutation that affects the tyrosine phosphorylation of the inhibitor had no effect on TGFβ1 activity. *P < 0.05, **P < 0.01, and ***P < 0.001.

Based on the changes observed in dendrite morphology, and in neuronal connectivity and survival following *Hes1 *overexpression, we investigated the effects of an alternative means of activating NF-κB using the cytokine TGFβ1 [22]. The role of TGFβ1 in neuronal polarity and axonal specification has been studied previously [23], and while TGFβ1-3 promote dendrite growth in retinal ganglion cells [24], the effects of TGFβ on neuronal plasticity remain unclear. We first studied the effects of TGFβ1 on dendritic patterning in cultured hippocampal neurons after 7 DIV. Exposure to TGFβ1 increased the number of primary dendrites while decreasing the number of remaining dendrites (Figure 4A). Moreover, GABAergic connectivity was augmented in cultured hippocampal neurons treated with TGFβ1, as revealed by VIAAT immunostaining (Figure 4A, right panel). Varicosities containing VIAAT also increased upon TGFβ1 administration. The effects of TGFβ1 were mediated by *Hes1 *as TGFβ1 had no such activity in neurons transfected with the *Hes1 *inhibitor *Hes6 *[19,20,25]. Impairment of *Hes1 *also prevented TGFβ1 from altering dendrite patterning and GABAergic connectivity.

Although the TGFβ1 transduction pathway that modulates neuronal plasticity is poorly understood (but see Discussion), TGFβ1 is known to activate NF-κB in hippocampal neurons [22]. We found that this activation of NF-κB by TGFβ1 may be involved in neuronal plasticity, and thus we analyzed the morphological changes induced by TGFβ1 in cells transfected with either a non-serine phosphorylatable mutant I-κBα or a non-tyrosine phosphorylatable form of I-κBα. Transfection with the serine mutant blocked the effects of TGFβ1 on dendrite elongation and GABAergic connectivity (Figure 4B), whereas transfection with the tyrosine mutant of I-κBα had no effect on TGFβ1 activity. Accordingly, the effects of TGFβ1 on neuronal plasticity are dependent upon serine phosphorylation of I-κB and its capacity to activate NF-κB. By contrast, overexpression of the tyrosine mutant form had no effect on TGFβ1 activity (Figure 4B). In conjunction with previous findings [12], these results suggest that *Hes1 *can be activated by either NGF or TGFβ1. Both these factors activate NF-κB, although the former degrades I-κBα by phosphorylation at tyrosine 42 [12] and the latter by phosphorylating serines 32 and 36.

### Anti-amyloid effects of TGFβ1

**Figure 5:**
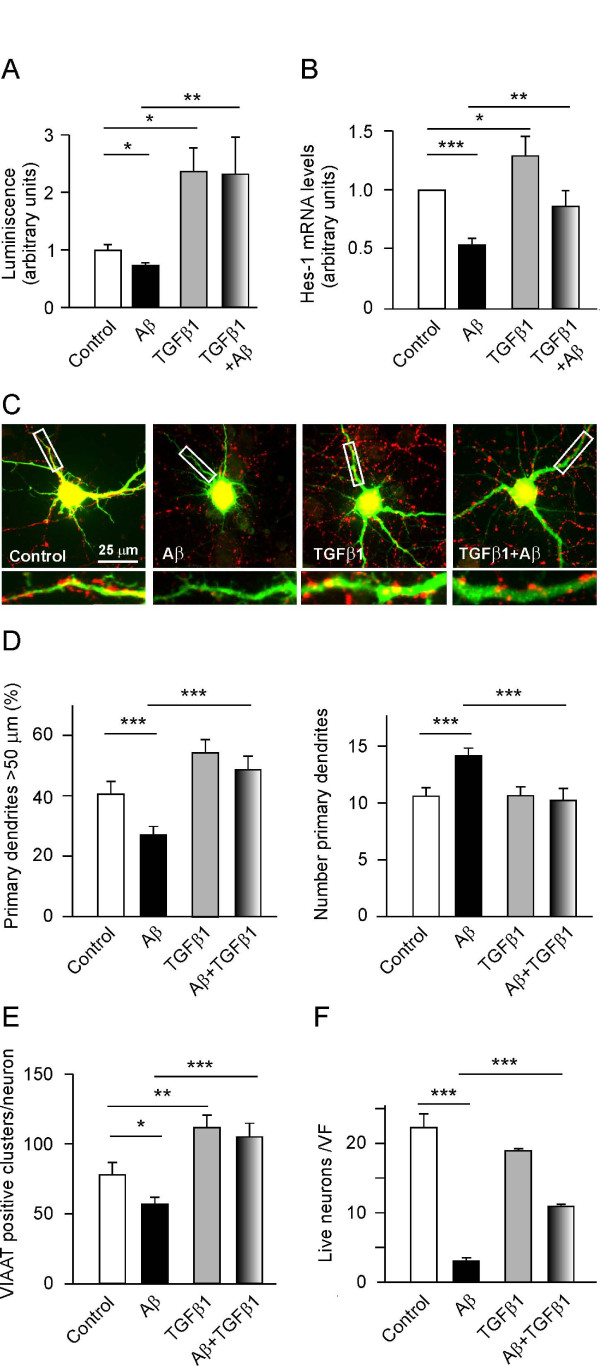
**Tranforming growth factor β1 (TGFβ1) activates nuclear factor kappa-B (NF-κB) and induces *Hes1 *expression, controlling dendrite morphology and GABAergic connectivity, and conferring cultured neurons with resistance to amyloid beta (Aβ) neurotoxicity**. (**A**) Dual-luciferase NF-κB reporter gene assay. After transfecting NF-κB-luc and RL-TK-luc, 7 days *in vitro *(DIV) hippocampal neurons were incubated for 16 h and the neurons were then exposed to Aβ (5 μM) with TGFβ1 (10 ng/ml) added to the medium 1 h later. The neurons were analyzed after a further 4 h incubation and the data represent the means of six experiments. (**B**) Cultured neurons (7 DIV, 300,000 cells) were treated with Aβ (5 μM) and then TGFβ1 (10 ng/ml) 1 h later, and the cells incubated for a further 16 h. After lysing the cells, *Hes1 *expression was quantified by real time PCR (the means of 10 determinations are shown). (**C-E**) Hippocampal neurons (7 DIV, 40,000 cells/cm^2^) were transfected with enhanced green fluorescent protein (EGFP) and treated with Aβ (5 μM) and/or TGFβ1 (10 ng/ml). After 16 h, immunostaining for EGFP and vesicular inhibitory amino acid transporter (VIAAT) was performed as described in Materials and methods. (**C**) Representative micrographs of cultured neurons under different conditions (the lower panels show the boxed regions at higher magnification). (**D**) Morphometric analysis of Aβ- and TGFβ1-treated neurons. TGFβ1 increased the length (left panel) and decreased the number (right panel) of primary dendrites, thereby counteracting the effects of Aβ. (**E**) Quantification of GABAergic clusters. TGFβ1 increased the number of GABAergic terminals in cultured neurons and overrode the decrease in GABAergic terminals induced by Aβ. (**F**) Hippocampal neurons (7 DIV, 30,000 cells/cm^2^) were treated with Aβ (5 μM) and/or TGFβ1 (10 ng/ml) for 90 h and the number of intact nuclei were counted after 4',6-diamidino-2-phenylindole (DAPI) staining. TGFβ1 prevented Aβ-induced death of a portion of cultured hippocampal neurons. *P < 0.05, **P < 0.01, and ***P < 0.001.

As Aβ prevents NGF-induced tyrosine phosphorylation and the subsequent degradation of I-κBα [12], we asked whether TGFβ1 could counteract the noxious effects of Aβ by activating NF-κB after having promoted serine phosphorylation of I-κBα. Using a reporter gene luminescent assay, we demonstrated that TGFβ1 activated NF-κB (Figure 5A), more than doubling its activity in cultured neurons. Moreover, the reporter gene assay revealed a modest but significant decrease in NF-κB activity produced by Aβ, which significantly failed to prevent TGFβ1-induced activation of NF-κB. In accordance with this observation, we found that TGFβ1 increased neuronal *Hes1 *mRNA expression, while Aβ induced a significant reduction in *Hes1 *expression (Figure 5B). Indeed, exposure to TGFβ1 partially restored the low levels of *Hes1 *expression induced by Aβ. These results demonstrate that TGFβ1 opposes the effects of Aβ on NF-κB activation and *Hes1 *expression.

Based on the above findings, we examined the anti-amyloid effects of TGFβ1 on neuronal morphology, connectivity and survival (Figure 5C-F). TGFβ1 blocked the effects of Aβ on dendrite length and number (Figure 5C, D), and it prevented the Aβ-induced decrease in the number of GABAergic terminals (Figure 5C, E). Finally, administration of TGFβ1 to cultured neurons protected about 50% of neurons from Aβ neurotoxicity Figure 5F). These results emphasize the potential of TGFβ1 as a neuroprotective agent and reveal, at least in part, the molecular basis of this neuroprotective activity.

## Discussion

### The effect of NF-κB activity on neuron survival

Transcription factors, including NF-κB, are implicated in experience-based synaptic regulation, and mouse models involving altered NF-κB activity have revealed the importance of the various forms of this transcription factor in learning and memory (reviewed in [26]). NF-κB may influence neuronal plasticity at multiple levels as it mediates neurite outgrowth [27] and participates in the development of dendritic spines [28]. NF-κB also plays an important role in the dendritic development of Purkinje cells, since when it is inhibited with a lysyl oxidase peptide, serious deficits in dendritic arborisation are provoked [29]. Here, we show that p65/RelA transfection induces significant changes in the morphology of the dendrites emitted by cultured hippocampal neurons. These alterations were similar to those previously described for NGF, the effects of which are partially mediated by NF-κB [13]. Overexpression of p65/RelA induces an increase in dendritic length and a decrease in dendrite number within 16 h. Perhaps more importantly, p65/RelA overexpression counteracts the effects of Aβ on dendrite morphology, decreasing dendrite length and increasing the number of primary dendrites. This finding provides an important indication of the anti-amyloid effects of p65/RelA overexpression.

NF-κB exerts neuroprotective effects against some neurotoxic agents [30,31], including Aβ [32], and total abrogation of NF-κB activation by pharmacological agents was followed by hippocampal neuron death (Figure S2A in Additional file 2). However, with the exception of bcl-XL induction [33] and the suppression of apoptotic proteins such as Bax and Bim [34], the mechanisms underlying NF-κB-induced neuroprotection remain elusive. In neurons, NF-κB is required to maintain high GluR1 levels and neuronal hyperexcitability following the induction of long-term potentiation (LTP) [35]. However, increases in NF-κB activity in response to enhanced excitatory transmission may accelerate the onset of the cognitive defect in a mouse model of Alzheimer's disease [36]. The present results show that p65/RelA promotes GABAergic connectivity in cultured hippocampal neurons, as revealed by the substantial increases in terminals containing VIAAT. We previously reported a substantial loss of VIAAT-labeled terminals shortly after Aβ administration in cultured hippocampal neurons [12] These effects were fully prevented by overexpressing p65/RelA, which may explain the basis underlying the anti-amyloid activity of NF-κB.

Together with inhibitors of NF-κB kinases, NF-κB regulates many physiological responses, and activation of IKK in neurons should induce similar cellular changes to those elicited by p65/RelA overexpression. The canonical pathway of NF-κB activation involves I-κBα phosphorylation via activation of the IKK complex (reviewed in [37]). Transfection of hippocampal neurons with a plasmid expressing IKKβ promoted dendritic growth while decreasing the number of primary dendrites. Furthermore, IKKβ transfection prevented Aβ from altering dendritic patterning. Most importantly, IKKβ overexpression protected a significant number of neurons from the deleterious effects of Aβ. Thus, canonical activation of NF-κB conferred amyloid resistance to cultured hippocampal neurons.

### The role of Hes1 in anti-amyloid neuroprotection

*Hes1 *is an important target of IKKβ/NF-κB in terms of its influence on neuronal morphology and survival. Activation of NF-κB by NGF increases *Hes1 *expression, whereas specific inhibition of this nuclear factor abrogates the activity of the neurotrophin [12,13] and it eventually caused cell death (Figure S2A in Additional file 2). We found that overexpression of either IKKβ or p65/RelA induces an increase in *Hes1 *expression in transfected hippocampal neurons. To further demonstrate that both the morphological and anti-amyloid effects of IKKβ and p65/RelA depend upon *Hes1 *expression, we transfected neurons with a vector that drives the overexpression of this gene. *Hes1 *overexpression induced the same morphological changes as seen following IKKβ or p65/RelA overexpression, including a large increase in GABAergic connectivity. As expected, *Hes1 *overexpression counteracted the effects of Aβ on cell morphology and GABAergic terminals, and strikingly, overexpression of *Hes1 *also rescued 50% of neurons from Aβ-induced death. Conversely, inhibition of *Hes1 *activity by overexpression of *Hes6 *induced cell death (Figure S2B in Additional file 2). Together, these findings indicate that correct expression of *Hes1 *confers anti-amyloid resistance to cultured hippocampal neurons, strongly suggesting that strategies to increase *Hes1 *expression and activity may protect neurons from Aβ toxicity.

### TGFβ1 provides an alternative means of promoting Hes1 expression and inducing anti-amyloid activity

TGFβ1 has long been recognized as a neuroprotective agent (for a review see [38]) and indeed, neurodegeneration [39,40] and Aβ deposition [41] are enhanced in TGFβ1-deficient mice. Furthermore, components of the hippocampal TGFβ pathway are altered in schizophrenia and psychiatric disorders [42]. Several TGFβ signalling pathways have been elucidated [43] and the canonical pathway involves the activation and nuclear localization of the Smad complex, where it modulates target gene transcription. However, our data suggest that the neuroprotective activity of TGFβ1 is not mediated by this canonical pathway but rather, by NF-κB/*Hes1*. Administration of TGFβ1 to cultured neurons alters dendritic patterning and GABAergic connectivity in a manner consistent with *Hes1 *overexpression. Moreover, transfection with *Hes6*, an inhibitor of *Hes1 *transcriptional activity [19,20,25], abrogated all the effects of TGFβ1 on neuronal morphology and connectivity. Although *Hes1 *upregulation by TGFβ1 has been reported previously in fibroblasts [44], this is the first time the regulation of this bHLH gene by TGFβ1 has been described in hippocampal neurons.

Further assays of neuronal morphology and connectivity revealed the involvement of I-κBα in TGFβ1 signalling. Transfection with a serine mutant form of I-κBα abolishes the effects of TGFβ1 on both dendritic shape and on the number of GABAergic terminals. However, a tyrosine mutant form of I-κBα had no effect on TGFβ1 activity, indicating that serine phosphorylation of I-κBα preceded NF-κB activation in this pathway. Direct biochemical measurements revealed that treatment of cultured neurons with TGFβ1 promoted NF-κB activation and *Hes1 *expression. The activation of NF-κB by TGFβ1 has been reported previously in cultured hippocampal neurons from rat embryos [22]. However, we also observed that TGFβ1 reversed the loss in NF-κB activity and *Hes1 *expression induced by Aβ. Indeed, TGFβ1 also prevented the formation of VIAAT-positive clusters in response to Aβ, and it prevented Aβ from altering dendrite patterning. Most importantly, TGFβ1 rescued a significant portion of neurons from Aβ-induced death. These findings confirm the neuroprotective capacity of this cytokine and the underlying role of NF-κB activation and *Hes1 *expression.

We showed previously that the action of Aβ is exerted in part by inhibiting early steps in the NGF signalling pathway, including the deactivation of RhoA and the activation of PTP1B, both events that are needed for NF-κB activation and to promote *Hes1 *expression [15]. Accordingly, TGFβ1 can circumvent such effects by activating NF-κB through an alternative mechanism that involves the serine phosphorylation of I-κB, as shown here. However, when NF-κB activation was pharmacologically impaired by SN-50 or when *Hes1 *activity was blocked by *Hes6 *overexpression, TGFβ1 activity was abrogated (Figure S2A and S2B in Additional file 2). Together, these results suggest that on the one hand, NF-κB activation and *Hes1 *activity are needed for cell survival, and on the other, that TGFβ1 exert its anti-amyloid activity by potentiating NF-κB and *Hes1 *activities.

### Modulation of GABAergic input in neuroprotection

Growth factors may control synaptic development and transmission in quantitative terms. Brain-derived neurotrophic factor (BDNF) upregulates glutamatergic input and downregulates the number of GABAergic synaptic terminals [45], while insulin promotes the postsynaptic accumulation of GABA_A _receptors by increasing Akt-mediated phosphorylation of β subunits (reviewed in [46]). NGF also increases the expression of GABAergic terminals in cultured hippocampal neurons, an effect mediated by altering *Hes1 *expression [13]. Via the canonical pathway involving Smad4, TGFβ1 is a critical factor in use-dependent modulation of GABA_A_-mediated synaptic transmission and dendrite homeostasis [47].

In all experimental paradigms assayed here, including the transfection of cultured neurons with IKKβ, Iκ-Bα or p65/RelA and the exposure of cultured neurons to TGFβ1, VIAAT immunocytochemistry indicated that there was a large increase in the expression of GABAergic terminals. These experimental approaches also consistently prevented Aβ from affecting GABAergic terminals, since exposure to Aβ decreases the number of GABAergic connections after 16 h and kills cells after 90 h. Thus, there is compelling evidence that *Hes1 *is an important element in the maintenance of GABAergic connectivity, although the mechanisms underlying this phenomenon remain unknown. The increase in GABAergic input promoted by NF-κB/*Hes1 *may provide a negative feedback in the control of excitatory activity and consequently, protect neurons from excitotoxicity. The increase in inhibitory activity induced by NF-κB activation or TGFβ1 administration may account for the neuroprotective effects observed. Indeed, TGFβ1 plays an important role in the excitatory/inhibitory balance of hippocampal transmission [47].

## Conclusions

**Figure 6:**
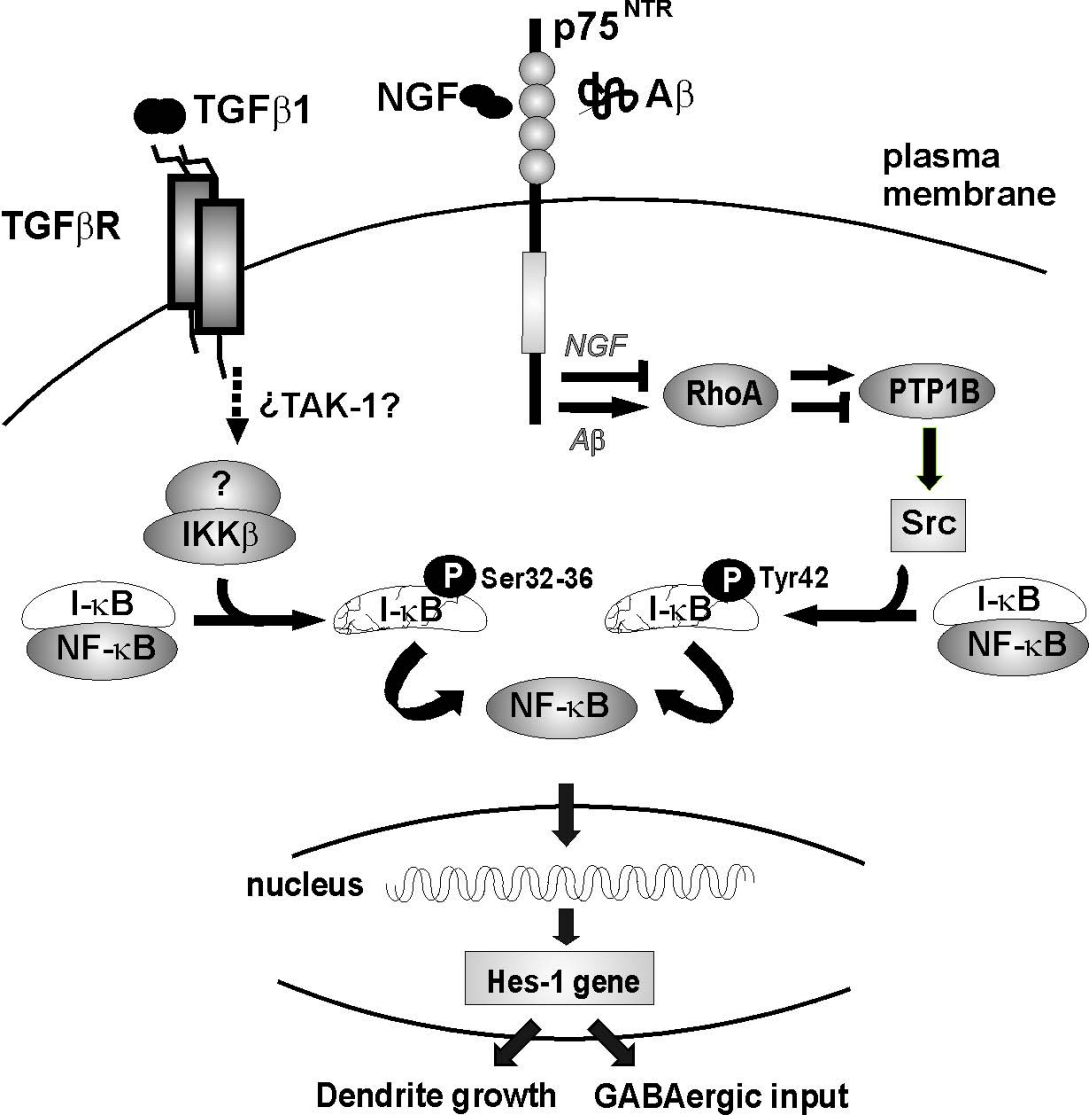
**Transforming growth factor β1 (TGFβ1), nerve growth factor (NGF) and amyloid beta (Aβ)-induced signaling in hippocampal neurons**. Diagram outlining the signal transduction pathways downstream of TGFβ1 that promote *Hes1 *expression and neuronal survival. How NGF is impaired by Aβ is also illustrated. In contrast to NGF, the activation of NF-κB and the increased expression of *Hes1 *induced by TGFβ1 are not inhibited by Aβ.

The findings presented here support the notion that neurons can be protected from the noxious effects of Aβ by modulating inhibitory transmission. Moreover, strategies that mildly activate NF-κB and/or that enhance *Hes1 *expression could provide beneficial neuroprotection. Significantly, TGFβ1 signalling counteracts the inhibitory effect of Aβ on NGF signalling (summarized in Figure 6), possibly supplementing the attenuated activity of NGF in Alzheimer's disease and representing a potential target for the development of anti-amyloid therapies.

## Abbreviations

Aβ: amyloid beta; AD: Alzheimer's disease; Akt: acutely transforming retrovirus AKT8 in rodent T cell lymphoma; BDNF: brain-derived neurotrophic factor; bHLH: basic helix-loop-helix; DAPI: 4',6-diamidino-2-phenylindole; DMSO: dimethyl sulfoxide; EGFP: enhanced green fluorescent protein; FAM: 6-carboxy fluorescein; GABA: gamma-aminobutyric acid; GLU1: glutamate receptor 1; HRP: horseradish peroxidise; I-κBα: I kappa Bα; Hes1: enhancer of split homolog 1; IKKB: I-κB kinase; LTP: long-term potentiation; NF-κB: nuclear factor kappa-B; NGF: nerve growth factor; PBS: phosphate-buffered saline; PFA: paraformaldehyde; PTP1B: protein tyrosine phosphatase 1B; PVDF: polyvinylidene difluoride; ROI: region of interest; TGFβ1: transforming growth factor β1; VIAAT: vesicular inhibitory amino acid transporter; wt: wild type.

## Competing interests

The authors declare that they have no competing interests.

## Authors' contributions

PJC and ART designed and performed research; PJC and ART analyzed data; PJC and ART wrote the paper. Both authors have read and approved the final manuscript.

## Supplementary Material

Additional file 1 **Figure showing molecular characterization and neurotoxic properties of the amyloid β (Aβ) preparations used in this study**. (**A**) The preparations of Aβ were characterized in western blots probed with an anti-Aβ antibody following Bis-Tris PAGE separation (see Methods). Lane (1) shows that the stock preparation mostly contained monomeric and dimeric species, with smaller quantities of trimeric and tetrameric forms. In lanes 2 to 5, Aβ was added to 35 mm culture dishes that containing one glass polylysine coated 2 × 2 cm coverslip and 2 mL of medium at the concentrations indicated. After a three-day incubation at 37°C, aliquots of the medium were taken and resolved by electrophoresis (supernatant, snt, lanes 2 and 3). Simultaneously, the glass coverslips were washed with LDS sample buffer and the material released was also separated in the same gels (immobilized on glass, imm, lanes 4 and 5). Note that the incubation of amyloid favoured the formation of higher molecular weight forms, although most species were small oligomers and the larger aggregates, including fibrils, only represented a small fraction of the amyloid. (**B**) Hippocampal neurons (7 days *in vitro *(DIV) and 30,000 cells/cm^2^) were treated with Aβ as indicated. After 90 h, the cells were fixed and stained with 4',6-diamidino-2-phenylindole (DAPI) to asses the integrity of their nuclei. Note that Aβ (5 μM) produced a high rate of cell death, which justified the use of this concentration in further experiments. VF, microscope view field. Click here for file

Additional file 2 **Figure showing the nuclear factor kappa B (NF-κB) pathway and *Hes1 *activity are needed for the survival of neurons, while transforming growth factor β1 (TGFβ1) is unable to rescue cells from death**. E17 hippocampal neurons were plated at a density of 30,000 cells/cm^2 ^and cultured for 7 days *in vitro *(DIV). Neurons were (**A**) treated for 24 h with SN-50 (5 μM) or with its control peptide in the presence or absence of TGFβ1 (10 ng/ml). (**B**) Neurons were co-transfected with enhanced green fluorescent protein (EGFP) and a myc-tagged *Hes6 *vector for 48 h in the presence or absence of TGFβ1. The cells were fixed and labeled with anti-EGFP and anti-myc antibodies, while the integrity of their nuclei was assessed by 4',6-diamidino-2-phenylindole (DAPI) staining. Note that the obliteration of either NF-κB activation or *Hes1 *activity was followed by neuron death. The addition of TGFβ1 did not reverse these effects. Click here for file
