# Supplementary figures and images for: Increased expression of the homologue of enhancer-of-split 1 protects neurons from beta amyloid neurotoxicity and hints at an alternative role for transforming growth factor beta1 as a neuroprotector
Source: Alzheimers Res Ther. 2012 Jul 31;4(4):31. doi: 10.1186/alzrt134 (PMC3506945; doi:10.1186/alzrt134)

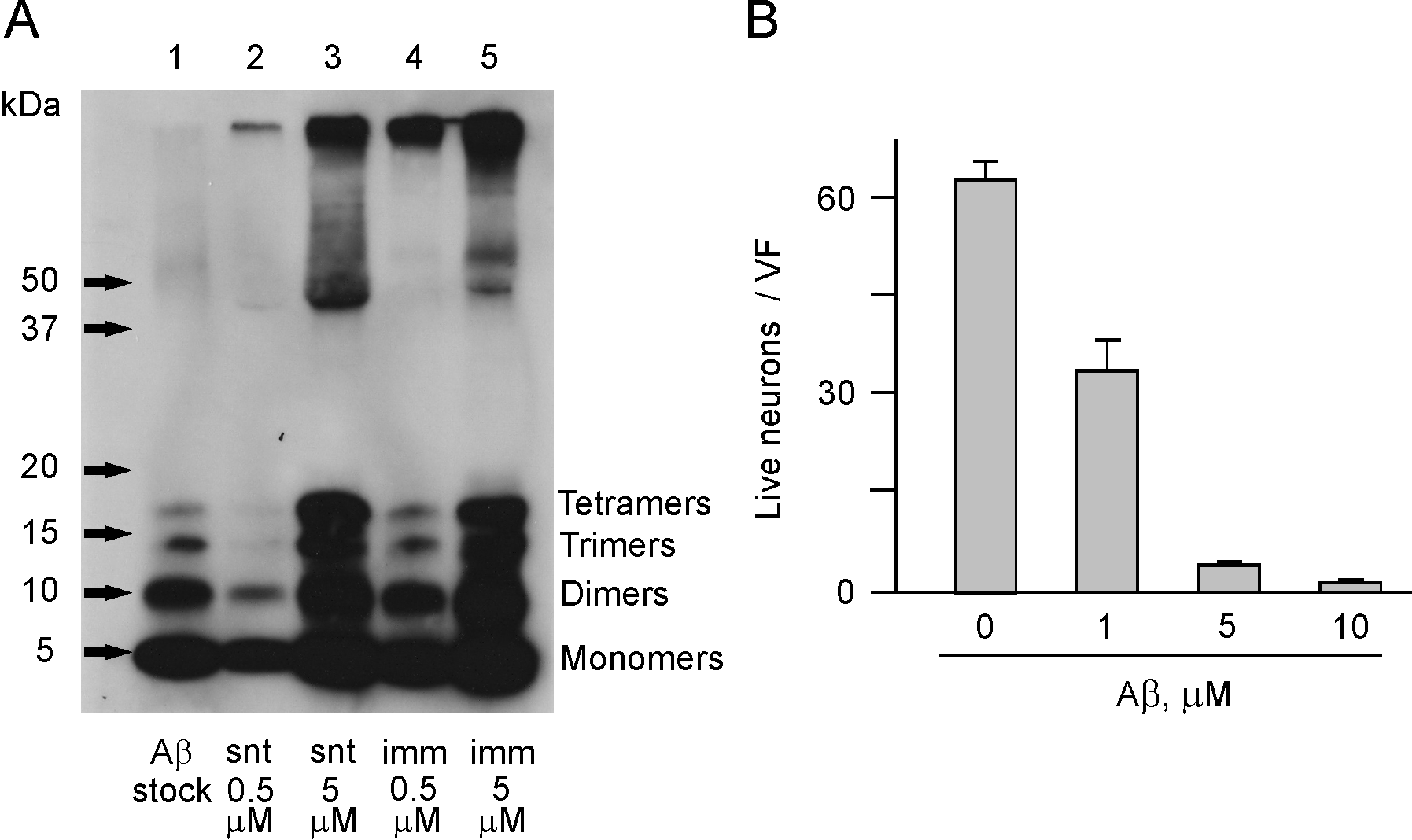

Supplement: Additional file 1 — Figure showing molecular characterization and neurotoxic properties of the amyloid β (Aβ) preparations used in this study. (A) The preparations of Aβ were characterized in western blots probed with an anti-Aβ antibody following Bis-Tris PAGE separation (see Methods). Lane (1) shows that the stock preparation mostly contained monomeric and dimeric species, with smaller quantities of trimeric and tetrameric forms. In lanes 2 to 5, Aβ was added to 35 mm culture dishes that containing one glass polylysine coated 2 × 2 cm coverslip and 2 mL of medium at the concentrations indicated. After a three-day incubation at 37°C, aliquots of the medium were taken and resolved by electrophoresis (supernatant, snt, lanes 2 and 3). Simultaneously, the glass coverslips were washed with LDS sample buffer and the material released was also separated in the same gels (immobilized on glass, imm, lanes 4 and 5). Note that the incubation of amyloid favoured the formation of higher molecular weight forms, although most species were small oligomers and the larger aggregates, including fibrils, only represented a small fraction of the amyloid. (B) Hippocampal neurons (7 days in vitro (DIV) and 30,000 cells/cm2) were treated with Aβ as indicated. After 90 h, the cells were fixed and stained with 4',6-diamidino-2-phenylindole (DAPI) to asses the integrity of their nuclei. Note that Aβ (5 μM) produced a high rate of cell death, which justified the use of this concentration in further experiments. VF, microscope view field. [file alzrt134-S1.TIFF]

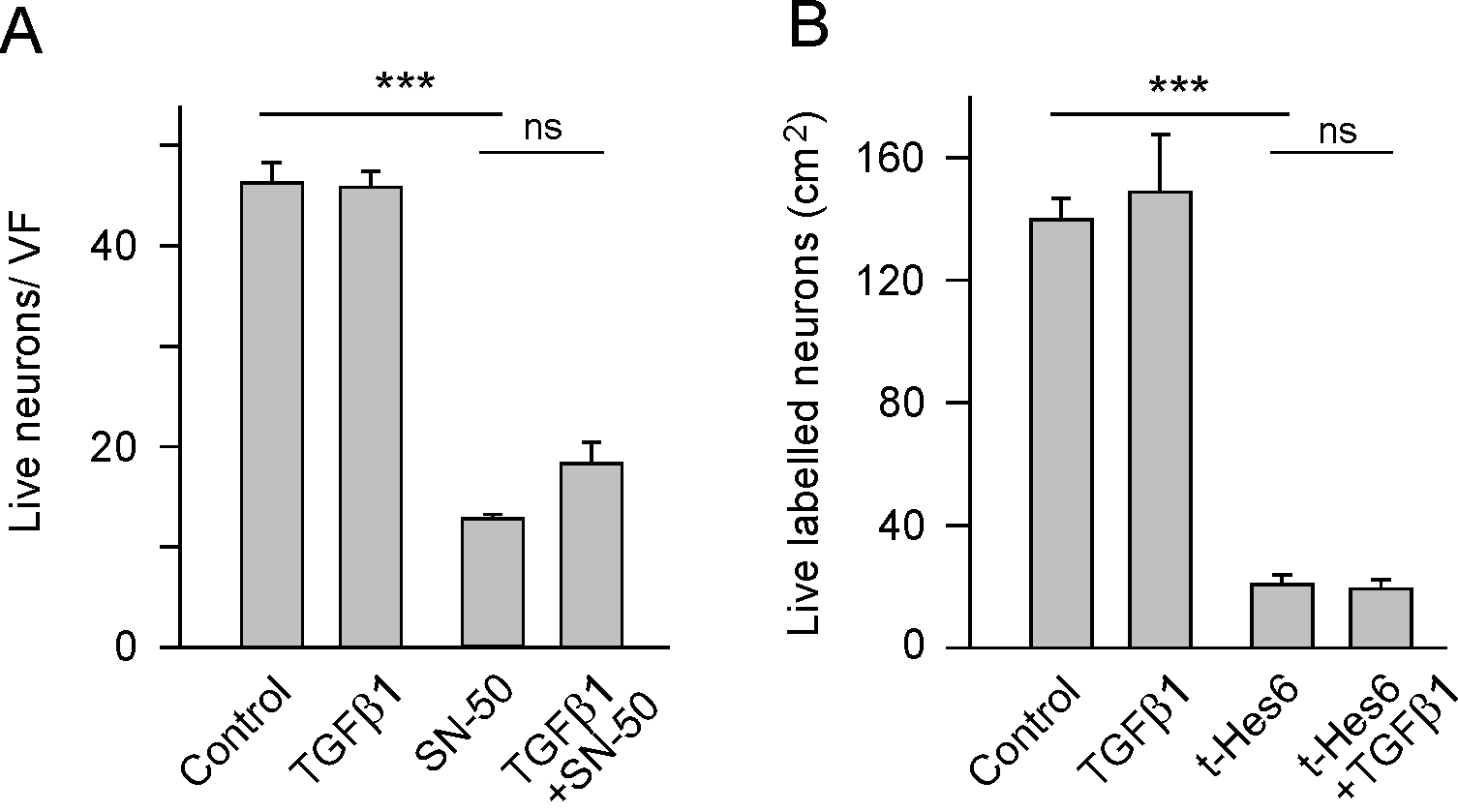

Supplement: Additional file 2 — Figure showing the nuclear factor kappa B (NF-κB) pathway and Hes1 activity are needed for the survival of neurons, while transforming growth factor β1 (TGFβ1) is unable to rescue cells from death. E17 hippocampal neurons were plated at a density of 30,000 cells/cm2 and cultured for 7 days in vitro (DIV). Neurons were (A) treated for 24 h with SN-50 (5 μM) or with its control peptide in the presence or absence of TGFβ1 (10 ng/ml). (B) Neurons were co-transfected with enhanced green fluorescent protein (EGFP) and a myc-tagged Hes6 vector for 48 h in the presence or absence of TGFβ1. The cells were fixed and labeled with anti-EGFP and anti-myc antibodies, while the integrity of their nuclei was assessed by 4',6-diamidino-2-phenylindole (DAPI) staining. Note that the obliteration of either NF-κB activation or Hes1 activity was followed by neuron death. The addition of TGFβ1 did not reverse these effects. [file alzrt134-S2.TIFF]
